# Identification and Analysis of the Porcine MicroRNA in Porcine Cytomegalovirus-Infected Macrophages Using Deep Sequencing

**DOI:** 10.1371/journal.pone.0150971

**Published:** 2016-03-04

**Authors:** Xiao Liu, Shan Liao, Zhiwen Xu, Ling Zhu, Fan Yang, Wanzhu Guo

**Affiliations:** 1 Animal Biotechnology Center, College of Veterinary Medicine, Sichuan Agricultural University, Ya’ an, 625014, China; 2 Liver Center and Gastrointestinal Division, Department of Medicine, Massachusetts, General Hospital, Harvard Medical School, Boston, MA, 02114, United States of America; 3 Key Laboratory of Animal Disease and Human Health, College of Veterinary Medicine, Sichuan Agricultural University, Ya’ an, 625014, China; Kunming University of Science and Technology, CHINA

## Abstract

Porcine cytomegalovirus (PCMV; genus *Cytomegalovirus*, subfamily *Betaherpesvirinae*, family *Herpesviridae*) is an immunosuppressive virus that mainly inhibits the immune function of T lymphocytes and macrophages, which has caused substantial damage in the farming industry. In this study, we obtained the miRNA expression profiles of PCMV-infected porcine macrophages via high-throughput sequencing. The comprehensive analysis of miRNA profiles showed that 239 miRNA database-annotated and 355 novel pig-encoded miRNAs were detected. Of these, 130 miRNAs showed significant differential expression between the PCMV-infected and uninfected porcine macrophages. The 10 differentially expressed pig-encoded miRNAs were further determined by stem-loop reverse-transcription polymerase chain reaction, and the results were consistent with the high-throughput sequencing. Gene Ontology analysis of the target genes of miRNAs in PCMV-infected porcine macrophages showed that the differentially expressed miRNAs are mainly involved in immune and metabolic processes. This is the first report of the miRNA transcriptome in porcine macrophages and an analysis of the miRNA regulatory mechanisms during PCMV infection. Further research into the regulatory mechanisms of miRNAs during immunosuppressive viral infections should contribute to the treatment and prevention of immunosuppressive viruses.

## Introduction

Porcine cytomegalovirus (PCMV) is a member of the genus *Cytomegalovirus*, subfamily *Betaherpesvirinae*, family *Herpesviridae*. It is an icosahedral virus with a double-stranded linear DNA genome. PCMV is now widely distributed around the world. China, Britain, the United States, Germany, and Japan have all confirmed the prevalence of this virus [1–4]. PCMV is an immunosuppressive virus, which mainly inhibits the immune function of T lymphocytes and macrophages, and always co-infects with porcine reproductive and respiratory syndrome virus [5]. The immune suppression caused by PCMV leads to secondary infections, and the pigs are often subsequently infected with *Bordetella bronchiseptica*, *Haemophilus parasuis*, *Streptococcus*, and *Pasteurella suis*. PCMV induces serious systemic infections in piglets and embryos and causes miscarriage in sows. Infected pigs show clinical symptoms of pneumonia, dysplasia, and viremia [3, 6].

In humans, organ transplantation is the primary treatment for end-stage organ function failure, but it is limited by the shortage of donors. Xenotransplantation is an effective solution to the shortage of tissues for human transplantation; however, xenotransplantation also brings a risk of cross-species infection to the human recipients. Owing to the high infection rate of PCMV in pigs, it has become a major threat to xenotransplantation [7, 8].

MicroRNAs (miRNAs) are small (18–24 nt), noncoding RNAs that are widely found in eukaryotes. In the processing of miRNAs, the miRNA initial products, called primary miRNAs (pri-miRNAs), are transcribed from miRNA genes in the genome, and then the pri-miRNAs are processed by Drosha, an RNase III enzyme, into 60–80-nt hairpin structures called precursor miRNAs (pre-miRNAs). Subsequently, the pre-miRNAs are transported to the cytoplasm by exportin-5 protein and cleaved by another RNase III enzyme, Dicer, into mature miRNAs [9–11].

These mature miRNAs can regulate the expression of target genes by binding to the coding region or untranslated regions, and they play an important regulatory role in organism growth, hematopoiesis, organogenesis, cell differentiation, apoptosis, proliferation, fat metabolism, cancer, plant hormone levels, and signal transduction [12–14].

The in-depth study of miRNA regulatory mechanisms has revealed that they are involved in regulation of the animal immune system and play a key role in fighting off infections. The Illumina high-throughput sequencing platform breaks through the limitations of previous expression profile research techniques and provides a new outlook for miRNA research. This platform enabled us to sequence specifically sized miRNAs directly and to discover and identify novel miRNAs. Currently, Northern blots, DNA microarrays, stem-loop reverse-transcription polymerase chain reaction (RT-PCR), and *in situ* hybridization are the main confirmation methods for miRNAs. Stem-loop RT-PCR is a sensitive and efficient method for the detection of miRNAs, so it has been widely used for this purpose and was used in this study to confirm the high-throughput sequencing results [15, 16].

According to the data from a miRNA database (miRBase 21.0, http://www.mirbase.org/), 28,645 hairpin precursor miRNAs (pre-miRNAs) have been found, expressing 35,828 mature miRNAs in 223 species. The majority of viral miRNAs are from the herpesvirus family, but no PCMV-encoded miRNAs have been reported [17]. Many miRNAs show significant tissue, cell, or physiological stage-specific expression profiles, but some miRNAs have been found to be ubiquitous [17–20]. A series of virally infected host miRNA expression profiles have been reported, including the miRNA profiles of human cytomegalovirus (HCMV)-infected human dermal fibroblast cells and a pseudorabies virus-infected porcine epithelial cell line, but no reports on the miRNA expression profile of a PCMV-infected host have been published [21, 22].

Recent research has shown that PCMV infection causes a significant change in the transcriptome of the main organs of the porcine central immune system and significantly suppresses immune-related signaling pathways [23], therefore, we hypothesize that PCMV affects the expression of downstream immune-related genes by regulating the expression level of specific miRNAs in macrophages, promoting latent infection and immune evasion. We thus conducted a comprehensive analysis of the miRNA expression profile of porcine macrophages infected with PCMV. We also predicted host targets of the pig-encoded miRNAs that were significantly differentially expressed (DE).

## Materials and Methods

### Virus and cells for high-throughput sequencing

PCMV Sichuan strain and porcine macrophages (provided by the Animal Biotechnology Center of Sichuan Agricultural University) were used in this study. Cell cultures were performed in Corning 25-cm^2^ culture bottles (Corning, Shanghai, China) containing modified RPMI-1640 nutrient solution (Thermo Fisher Scientific, Waltham, UK) supplemented with 10% fetal bovine serum and 50 mg/ml penicillin/streptomycin antibiotic solution (Gibco, Beijing, China), and were maintained under standard conditions of 5% CO_2_ and 37°C. Porcine macrophages were infected with PCMV at 10 PFU per cell, and then PCMV-infected and uninfected porcine macrophages were collected at 72 h postinfection (hpi). Our study used pooled samples and experiments were performed in triplicate. All infected and control samples were stored until use in a single RNA isolation assay.

### RNA isolation

The total RNA from the PCMV-infected and uninfected porcine macrophages described above was extracted using Trizol reagent (Invitrogen, Shanghai, China), in accordance with the manufacturer’s instructions. The purity and concentration (ratio OD_260_/OD_280_) of total RNA samples were determined with a NanoDrop ND-1000 spectrophotometer (Nano Drop Inc., Wilmington, DE, USA).

### Data sources

The complete genomic sequence of PCMV is available in the NCBI GenBank Genome database (http://www.ncbi.nlm.nih.gov/nuccore/KF017583.1) (accession no.: KF017583.1). The annotated pig-encoded miRNAs are available in miRBase (http://www.mirbase.org/cgi-bin/query.pl?terms=ssc-miR) and Ensembl database (http://www.ensembl.org/index.html). The *Sus scrofa* genomic sequence, gene annotation information, and repeat sequences are available in the University of California Santa Cruz (UCSC) Genome Browser (http://genome.ucsc.edu/index.html).

### Small RNA library construction and sequencing

The total RNA from each sample was sequentially ligated to 3′ and 5′ small RNA adapters. cDNA was then synthesized and amplified using Illumina’s proprietary RT primers and amplification primers. Subsequently, 120–140-bp PCR-amplified fragments were extracted and purified from the PAGE gel. Next, the completed libraries were quantified using an Agilent 2100 Bioanalyzer. The samples were diluted to a final concentration of 8 pM, and cluster generation was performed on an Illumina cBot using a TruSeq Rapid SR cluster kit (#GD-402-4001, Illumina), following the manufacturer’s instructions. Finally, high-throughput sequencing was performed on an Illumina HiSeq 2000 using TruSeq Rapid SBS Kits (#FC-402-4002, Illumina), in accordance with the manufacturer’s instructions [24, 25].

### miRNA-seq data analysis

After sequencing, the total raw sequencing reads were filtered using the CHASTITY quality control filter. The clean reads that passed quality filtering were then further filtered by searching for reads whose 3′-ends aligned to at least 6 nt of the 3′-adapter. The adapter sequences were trimmed and the adapter-trimmed reads (≥15 nt) were aligned to the known *S*. *scrofa* pre-miRNA sequences in miRBase 19 (http://www.mirbase.org/) using Novoalign software (v2.07.11) with at most one mismatch. The unmapped reads and longer reads (length >15 nt) were discarded as unusable. Any sequences that matched the mature miRNA region, ±4 nt within a known pre-miRNA hairpin, were grouped and the 5-prime (5p) or 3-prime (3p) arms of the precursor hairpin were also indicated. The RNA sequence reads of rRNA, snRNA, tRNA, and snoRNA were excluded [26].

To correct for the difference in tag counts between samples, the tag counts were scaled to tpm (the copy number of transcripts per million) based on the total number of tags aligned to known *S*. *scrofa* pre-miRNAs in miRBase 19.

To predict novel pig-encoded and virus-encoded miRNAs, the miRDeep2 web server (http://www.mdc-berlin.de/en/research/research_teams/systems_biology_of_gene_regulatory_elements/projects/miRDeep/) was used. The fold-back secondary structures of the pig-encoded miRNAs were predicted using Mfold software (http://mfold.rna.albany.edu/). We arranged all the sequence data from the 3′ adapter-trimmed files, and the adapter-trimmed sequences whose length was <17 bp or had mismatches >1 bp were excluded. The significantly DE miRNAs for the infected sample *vs*. the uninfected sample were determined by fold change filtering. (A fold change ≥2.0 indicates that the specific miRNA in a PCMV-infected sample is upregulated, while a fold change ≤0.5 indicates that the specific miRNA in a PCMV-infected sample is downregulated.)

### Target prediction and functional enrichment of DE miRNAs

The database microT-CDS (http://diana.imis.athena-innovation.gr/DianaTools/index.php?r=microT_CDS/index), the positional relationships between miRNAs, and genomic annotation sets were used for the prediction of targets of the 130 DE miRNAs [27]. The target genes of the DE miRNAs were evaluated using Gene Ontology (GO). The GO project’s (http://www.geneontology.org) online server was used to provide a controlled vocabulary of terms for describing gene product characteristics and gene product annotation data from GO consortium members. We used Fisher’s exact test to determine whether there was more overlap between the DE list and the GO annotation list than would be expected by chance. The *p*-value denotes the significance of the enrichment of GO terms in the DE genes. The GO terms with *p* < 0.05 were considered significant. The WEGO software (http://wego.genomics.org.cn/cgi-bin/wego/index.pl) was used to produce histograms of the GO annotations [28].

### Stem-loop RT-PCR

The total RNA from each sample was extracted using Trizol reagent (Invitrogen), in accordance with the manufacturer’s instructions. After RNA had been reverse-transcribed using an RT-PCR kit (GeneCopoeia, Guangzhou, China), the RT products were amplified using specific primers (Table 1), and the amplification conditions were as follows: 94°C for 3 min, followed by 40 cycles of 95°C for 20 s, 60°C for 40 s, and 72°C for 20 s, with a final elongation at 72°C for 5 min. RT-PCR reactions were carried out using the SYBR Green PCR Core Reagents Kit (Applied Biosystems, Foster City, CA, USA) on an ABI Prism 7900 Sequence Detection System (Applied Biosystems), in accordance with the manufacturer’s instructions.

**Table 1:** Primers used for RT-PCR.

Gene	Primers	Production length (bp)
U6	F:5’ TCGCTTTGGCAGCACCTAT 3’ R:5’ AATATGGAACGCTTCGCAAA 3’	100
ssc-miR-24-3p	GSP:5’ TGGCTCAGTTCAGCAGGAACAG 3’ R:5’ GTCGGTGTCGTGGAGTCG 3’	76
ssc-miR-19b	GSP:5’ TGTGCAAATCCATGCAAAACTGA 3’ R:5’ GTCGGTGTCGTGGAGTCG 3’	77
ssc-miR-101	GSP:5’ TACAGTACTGTGATAACTGAA 3’ R:5’ GTCGGTGTCGTGGAGTCG 3’	75
ssc-miR-7	GSP:5’ TGGAAGACTAGTGATTTTGTTGTT 3’ R:5’ GTCGGTGTCGTGGAGTCG 3’	78
ssc-miR-128	GSP:5’ TCACAGTGAACCGGTCTCTTT 3’ R:5’ GTCGGTGTCGTGGAGTCG 3’	75
ssc-miR-155-5p	GSP:5’ TTAATGCTAATTGTGATAGGGG 3’ R:5’ GTCGGTGTCGTGGAGTCG 3’	76
ssc-miR-196b-5p	GSP:5’ TAGGTAGTTTCCTGTTGTTGGG 3’ R:5’ GTCGGTGTCGTGGAGTCG 3’	76
ssc-miR-18a	GSP:5’ TAAGGTGCATCTAGTGCAGATA 3’ R:5’ GTCGGTGTCGTGGAGTCG 3’	76
ssc-miR-novel-chr6_30729	GSP:5’ GGTAATACTGCCTGGTAATGATGA 3’ R:5’ GTCGGTGTCGTGGAGTCG 3’	76
ssc-miR-novel-chr5_29857	GSP:5’ GCAGCAGGTCTCCAAGGG 3’ R:5’ GTCGGTGTCGTGGAGTCG 3’	73

## Results

### Overview of the high-throughput sequencing data

PCMV is an important immunosuppressive virus; however, an understanding of its immune escape mechanisms is still lacking. To investigate the miRNA transcriptome in PCMV-infected cells, as well as to clarify the regulatory mechanisms of miRNAs during PCMV infection, we used a high-throughput sequencing technology on libraries of small RNAs from PCMV-infected and uninfected porcine macrophages. In this study, 3,987,148 clean reads and 3,575,737 adapter-trimmed reads (reads that have passed quality filtering, adapter filtering, and length filtering with length ≥15 nt) were generated by uninfected porcine macrophages, and 7,567,884 clean reads and 7,295,330 adapter-trimmed reads were generated by infected cells (NCBI GEO Accession number: GSE76156). Of these, 5,543,830 and 2,215,640 reads from PCMV-infected and uninfected porcine macrophages, respectively, aligned to known *S*. *scrofa* pre-miRNAs in miRBase 19 and the Ensembl database.

**Fig 1:**
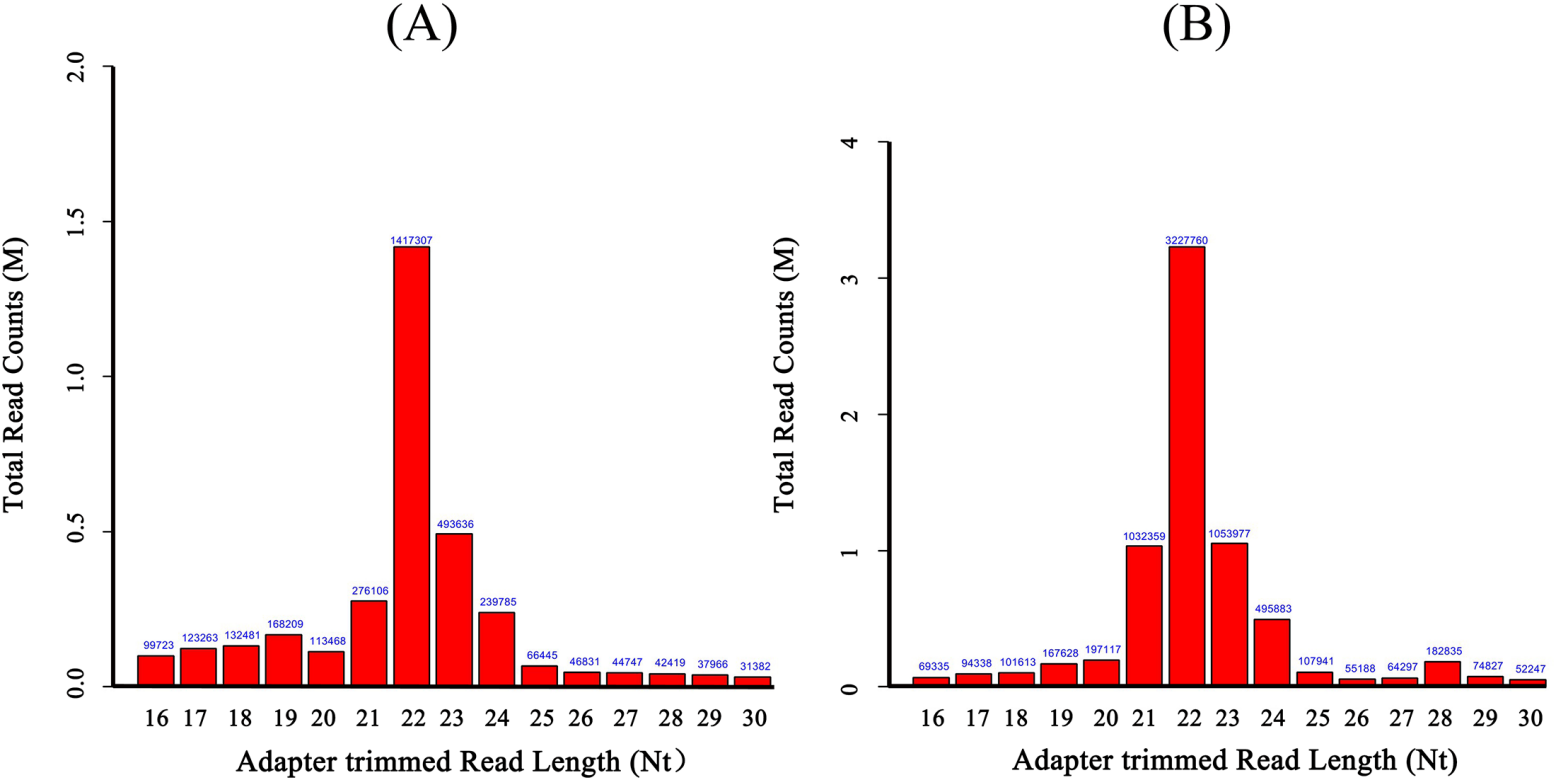
Length distribution of total sRNAs in PCMV-infected and non-infected porcine macrophages. (A) Length distribution of total sRNAs in uninfected porcine macrophages. (B) Length distribution of total sRNAs in PCMV-infected porcine macrophages.

The size distributions of the adapter-trimmed reads were similar in PCMV-infected and uninfected porcine macrophage libraries, and most of the reads were 22 nt in length (Fig 1). In addition to miRNAs, a series of noncoding small RNAs (sRNAs) were also detected in PCMV-infected and uninfected samples.

### Analysis of the pig-encoded miRNA expression profile

All filtered data were processed using Illumina’s Genome Analyzer Pipeline. When these data were aligned with the *S*. *scrofa* reference genome, 594 miRNAs were detected. Of these, 239 mature miRNAs were already annotated in miRBase 19, and 355 novel pig-encoded miRNAs were discovered in this study (S1 Table).

### Differential expression of porcine macrophage miRNAs

**Fig 2:**
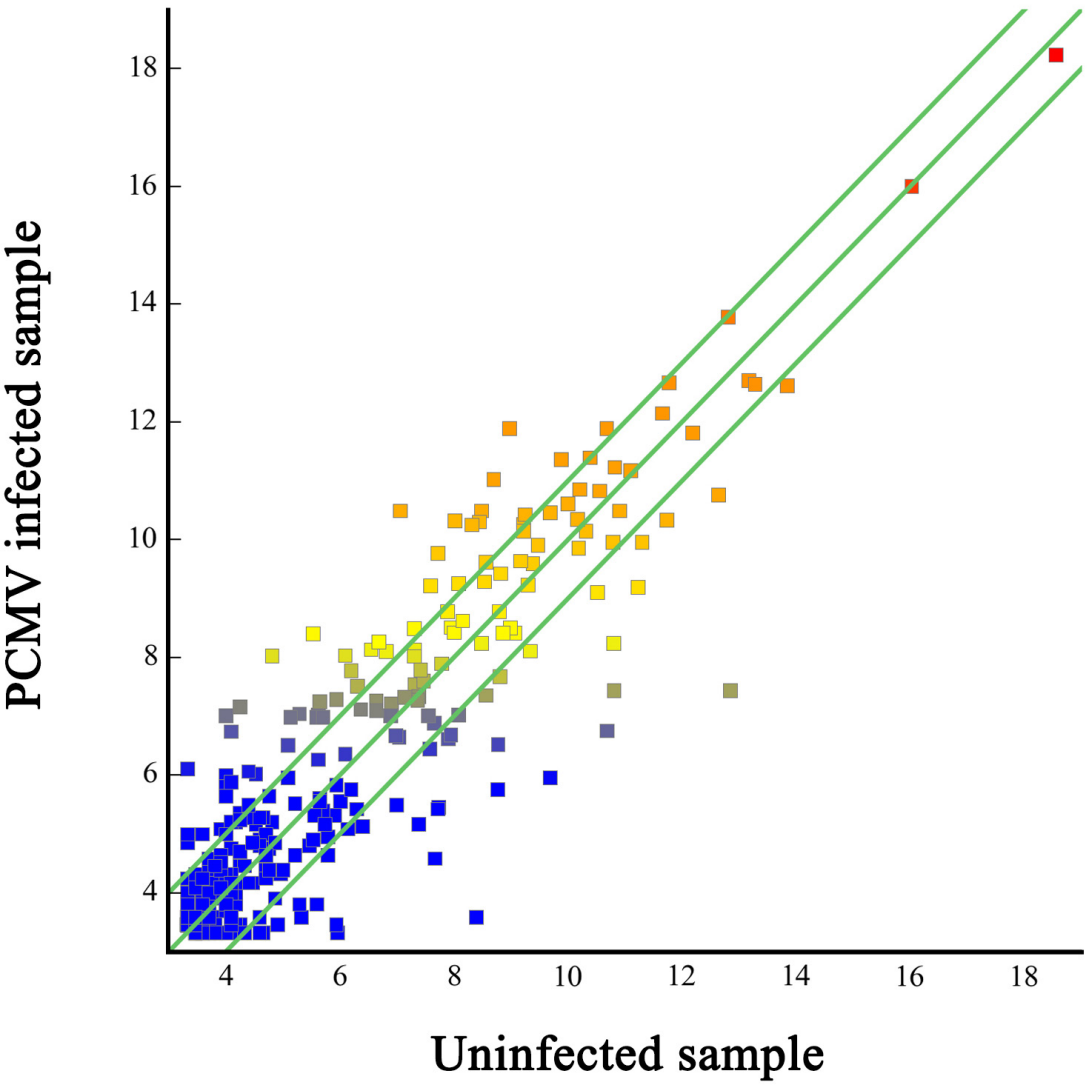
Scatter plot of differentially expressed miRNAs. The differentially expressed miRNAs are graphed on the scatter plot to visualize variations in miRNA expression between PCMV infected (values on the X axes) and uninfected macrophages (values on the Y axes). The default fold-change value is 2 (green lines).

**Fig 3:**
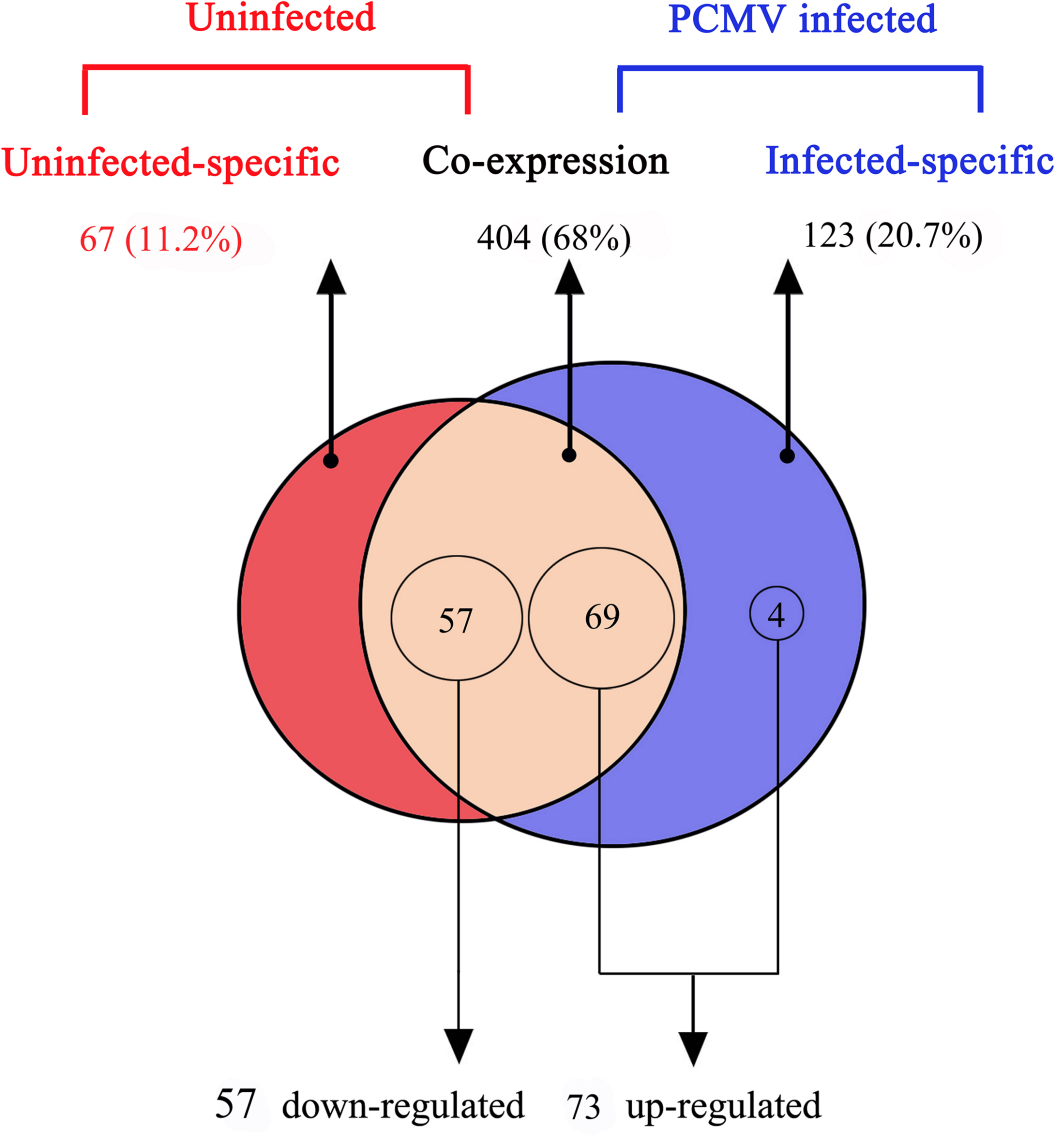
Comparison of differentially-expressed miRNAs between the PCMV-infected and uninfected porcine macrophages (*p<*0.0001). The Venn diagram shows the distribution of 594 unique miRNAs between uninfected (left, red circle) and PCMV-infected sample (right, blue circle) libraries. The overlapping section represents 404 co-expressed miRNAs. 130 miRNAs were significantly differentially expressed (*p<*0.0001).

Of the 594 detected miRNAs, 404 (68%) were coexpressed in PCMV-infected and uninfected porcine macrophages; however, 123 (20.7%) and 67 (11.2%) were expressed in the PCMV-infected and uninfected porcine macrophages, respectively (S2 Table, Table 2). The data on the differential expression of porcine macrophage miRNAs are displayed in a scatter plot (Fig 2). The deep sequencing data also showed that 130 unique pig-encoded miRNAs were significantly DE between the uninfected and PCMV-infected samples. Of these 130 miRNAs, 73 (56.2%) were significantly upregulated in PCMV-infected samples, while 57 (43.8%) were downregulated in them (Fig 3).

**Table 2:** Top 70 differentially expressed miRNAs in PCMV infected macrophages.

Mature miRNA	Precursors	Type	miRNA length	Fold-change value
ssc-miR-1	ssc-mir-1	Up-regulated	21	3.04
ssc-miR-101	ssc-mir-101-1	Up-regulated	21	18.60
ssc-miR-10a-3p	ssc-mir-10a	Up-regulated	21	3
ssc-miR-125b	ssc-mir-125b-1	Up-regulated	22	2.07
ssc-miR-128	ssc-mir-128-2	Up-regulated	21	4.97
ssc-miR-1307	ssc-mir-1307	Up-regulated	22	4.72
ssc-miR-139-5p	ssc-mir-139	Up-regulated	22	6.39
ssc-miR-142-3p	ssc-mir-142	Up-regulated	22	4.07
ssc-miR-143-3p	ssc-mir-143	Up-regulated	21	3.01
ssc-miR-148a-3p	ssc-mir-148a	Up-regulated	22	3.10
ssc-miR-148b-3p	ssc-mir-148b	Up-regulated	22	2.33
ssc-miR-155-5p	ssc-mir-155	Up-regulated	22	19.67
ssc-miR-192	ssc-mir-192	Up-regulated	21	2.45
ssc-miR-194a	ssc-mir-194a	Up-regulated	21	3.43
ssc-miR-194b-5p	ssc-mir-194b	Up-regulated	22	9.05
ssc-miR-196b-5p	ssc-mir-196b-1	Up-regulated	22	3.56
ssc-miR-20a	ssc-mir-20a	Up-regulated	22	3.03
ssc-miR-210	ssc-mir-210	Up-regulated	22	10.81
ssc-miR-215	ssc-mir-215	Up-regulated	21	7.39
ssc-miR-27b-3p	ssc-mir-27b	Up-regulated	21	2.82
ssc-miR-29c	ssc-mir-29c	Up-regulated	22	2.10
ssc-miR-30c-3p	ssc-mir-30c-2	Up-regulated	22	7.37
ssc-miR-32	ssc-mir-32	Up-regulated	21	2.82
ssc-miR-361-3p	ssc-mir-361	Up-regulated	24	3.38
ssc-miR-378	ssc-mir-378-1	Up-regulated	22	2.81
ssc-miR-7	ssc-mir-7-1	Up-regulated	24	7.60
ssc-miR-744	ssc-mir-744	Up-regulated	22	2.44
ssc-miR-769-5p	ssc-mir-769	Up-regulated	21	2.07
ssc-miR-99a	ssc-mir-99a	Up-regulated	22	3.85
ssc-miR-99b	ssc-mir-99b	Up-regulated	22	2.08
ssc-miR-novel-chr12_8265	ssc-mir-novel-chr12_8265	Up-regulated	20	2.56
ssc-miR-novel-chr15_15523	ssc-mir-novel-chr15_15523	Up-regulated	22	3.85
ssc-miR-novel-chr16_16917	ssc-mir-novel-chr16_16917	Up-regulated	21	3.02
ssc-miR-novel-chr2_21624	ssc-mir-novel-chr2_21624	Up-regulated	20	3.01
ssc-miR-novel-chr5_29674	ssc-mir-novel-chr5_29674	Up-regulated	21	2.37
ssc-miR-novel-chr5_29676	ssc-mir-novel-chr5_29676	Up-regulated	23	4.17
ssc-miR-novel-chr6_30729	ssc-mir-novel-chr6_30729	Up-regulated	22	2.02
ssc-miR-novel-chr6_30857	ssc-mir-novel-chr6_30857	Up-regulated	19	2.20
ssc-miR-novel-chr6_31604	ssc-mir-novel-chr6_31604	Up-regulated	21	3.63
ssc-miR-novel-chr7_33975	ssc-mir-novel-chr7_33975	Up-regulated	22	5.94
ssc-miR-novel-chr9_38959	ssc-mir-novel-chr9_38959	Up-regulated	17	3.04
ssc-miR-novel-chrX_40252	ssc-mir-novel-chrX_40252	Up-regulated	22	2.32
ssc-let-7c	ssc-let-7c	Down-regulated	22	0.47
ssc-let-7d-5p	ssc-let-7d	Down-regulated	22	0.10
ssc-let-7g	ssc-let-7g	Down-regulated	22	0.38
ssc-miR-146a-5p	ssc-mir-146a	Down-regulated	22	0.17
ssc-miR-15a	ssc-mir-15a	Down-regulated	21	0.22
ssc-miR-15b	ssc-mir-15b	Down-regulated	22	0.08
ssc-miR-185	ssc-mir-185	Down-regulated	22	0.07
ssc-miR-18a	ssc-mir-18a	Down-regulated	22	0.13
ssc-miR-193a-5p	ssc-mir-193a	Down-regulated	22	0.21
ssc-miR-195	ssc-mir-195	Down-regulated	21	0.12
ssc-miR-19a	ssc-mir-19a	Down-regulated	23	0.27
ssc-miR-19b	ssc-mir-19b-1	Down-regulated	23	0.43
ssc-miR-22-3p	ssc-mir-22	Down-regulated	22	0.44
ssc-miR-2320-5p	ssc-mir-2320	Down-regulated	22	0.41
ssc-miR-23a	ssc-mir-23a	Down-regulated	21	0.43
ssc-miR-24-3p	ssc-mir-24-1	Down-regulated	22	0.01
ssc-miR-28-5p	ssc-mir-28	Down-regulated	22	0.22
ssc-miR-31	ssc-mir-31	Down-regulated	22	0.21
ssc-miR-340	ssc-mir-340-1	Down-regulated	22	0.41
ssc-miR-361-5p	ssc-mir-361	Down-regulated	22	0.16
ssc-miR-423-5p	ssc-mir-423	Down-regulated	23	0.40
ssc-miR-542-3p	ssc-mir-542	Down-regulated	22	0.38
ssc-miR-98	ssc-mir-98	Down-regulated	22	0.24
ssc-miR-novel-chr11_6750	ssc-mir-novel-chr11_6750	Down-regulated	18	0.02
ssc-miR-novel-chr2_21617	ssc-mir-novel-chr2_21617	Down-regulated	17	0.17
ssc-miR-novel-chr5_29857	ssc-mir-novel-chr5_29857	Down-regulated	19	0.01
ssc-miR-novel-chr6_31474	ssc-mir-novel-chr6_31474	Down-regulated	17	0.09
ssc-miR-novel-chrX_39952	ssc-mir-novel-chrX_39952	Down-regulated	22	0.12

The fold change cutoffs of the upregulated miRNAs and the downregulated miRNAs were 2 and 0.5.

### Target prediction and gene functional annotation

To explore the regulatory roles of the DE miRNAs from this study, web prediction programs were used to predict their putative target genes (S3 Table). The miRNA regulatory network map generated by the web prediction programs showed that multiple miRNAs share the same target genes (S1 Fig). There were also many miRNAs targeting immune-related genes. For example, ssc-miR-19a targets TNFα, IL-12α, and IL-20; ssc-miR-19b targets TNFα and IL-20; ssc-miR-7 targets IL-1β, IL-2, and TGF-β1; ssc-let-7a targets IFN-delta-1; ssc-miR-30a-5p targets IL-12α and IL-12β; and ssc-miR-24-3p targets IL-1α.

**Fig 4:**
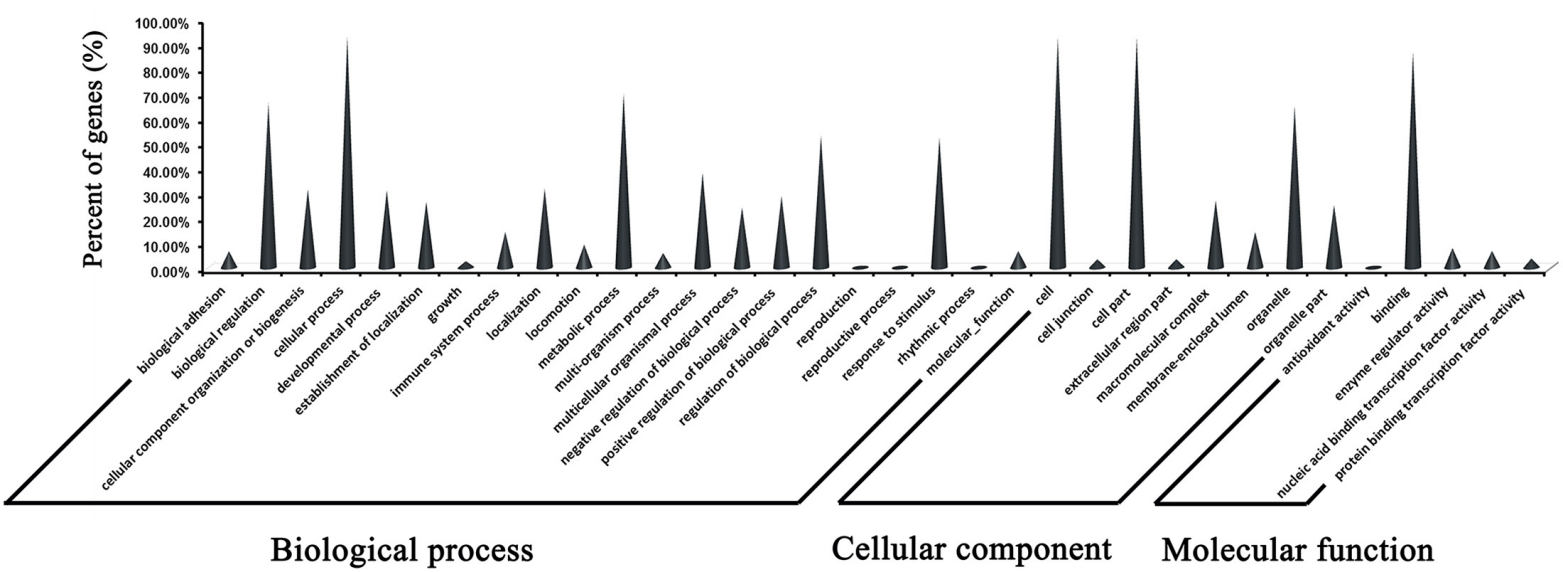
GO annotation of host target genes of differentially-expressed miRNAs. The host target genes of differentially-expressed miRNAs, annotated with their likely functions.

The results of the GO annotation reflected the regulatory roles that the pig-encoded miRNAs play in host physiological processes, including the regulation of cellular components, molecular functions, and biological processes. The target genes of the 130 DE miRNAs are mainly related to biological regulation of cellular, metabolic, and immune system processes (Fig 4, S4 Table). Of the target genes of DE miRNAs, 14.4% are related to immune system processes, which suggests that the changes in the miRNA expression profile during viral infection may affect immune function.

### Confirmation of the relative abundance of pig-encoded miRNAs

To confirm the relative abundance of pig-encoded miRNAs in PCMV-infected and uninfected porcine macrophages, we designed stem-loop real-time PCR primers to detect 10 DE miRNAs. We then quantified the expression level of these miRNAs, and the results obtained via RT-PCR were highly consistent with the high-throughput sequencing data (Table 3, S5 Table).

**Table 3:** Stem-loop RT-qPCR confirmation for the significantly differentially-expressed miRNAs.

Mature miRNA ID	Pre-miRNA ID	qRT-PCR fold-change (Infected/control)	High throughput sequencing fold-change (Infected/control)
ssc-miR-101	ssc-mir-101-1	+1.48	+18.60
ssc-miR-7	ssc-mir-7-2	+2.69	+7.60
ssc-miR-128	ssc-mir-128-1	+1.55	+4.97
ssc-miR-155-5p	ssc-mir-155	+3.03	+19.67
ssc-miR-196b-5p	ssc-mir-196b-1	+1.36	+3.56
ssc-miR-novel-chr6_30729	ssc-mir-novel-chr6_30729	+1.51	+2.02
ssc-miR-18a	ssc-mir-18a	-0.34	-0.13
ssc-miR-19b	ssc-mir-19b-2	-0.98	-0.43
ssc-miR-24-3p	ssc-mir-24-1	-0.29	-0.01
ssc-miR-novel-chr5_29857	ssc-mir-novel-chr5_29857	-0.44	-0.015

Comparison of the levels of miRNA expression detected by qRT-PCR and high-throughput sequencing. “+” and “−” indicate upregulated and downregulated miRNAs, respectively. The fold change cutoffs of the upregulated miRNAs and the downregulated miRNAs were 2 and 0.5, respectively. qRT-PCR Ct threshold is 0.015.

## Discussion

Similar to HCMV, PCMV also belongs to the herpes virus family and has the biological characteristic of latency activation [5]. Because of the high homology between human and pig genomes, further research on PCMV should also contribute to the prevention and treatment of HCMV [1]. Additionally, PCMV is the major threat to pig—human xenotransplantation, and the immunosuppressive therapy necessary after surgery may aggravate an infection of PCMV; therefore, further research into the molecular mechanisms of PCMV infection should contribute to the development of xenotransplantation technology [29].

To better understand the roles of miRNAs in immune suppression mechanisms and pathological processes, a new-generation high-throughput sequencing platform (Illumina HiSeq 2000) was used in this study to determine the miRNA transcriptome in PCMV-infected cells. Unfortunately, PCMV-encoded miRNAs were not detected in this study, so whether PCMV-encoded miRNAs exist in infected hosts needs further confirmation.

In this study, PCMV-infected porcine macrophages were used as a model for researching the regulatory function of miRNAs during the infection of an immunosuppressive virus, and a series of mammalian-encoded miRNAs were detected. Under the stress of PCMV infection, 73 miRNAs were upregulated and 57 miRNAs were downregulated in porcine macrophages compared with their expression levels in the uninfected group, of which four specific upregulated miRNAs (ssc-miR-424-3p, ssc-miR-novel-chr15_16769, ssc-miR-novel-chr16_17559, ssc-miR-novel-JH118654-1_42487) were expressed only in PCMV-infected porcine macrophages. This shows that virally infected cells might affect the expression of immune-related miRNA target genes through regulating the expression level of specific miRNAs, thereby regulating the immune process.

The miRNA expression level is known to be significantly reduced in cancer tissue relative to that in healthy tissue [30], but in the present study, the miRNA expression level was significantly increased after PCMV infection (adapter-trimmed reads of PCMV-infected and uninfected macrophages numbered 3,575,737 and 7,295,330, respectively). This suggests that the host miRNA expression profile was affected by viral infection. Interestingly, viral infection in porcine macrophages triggered the significant differential expression of some specific miRNAs, which has also been confirmed in other studies [31, 32]. This indicates that these specific miRNAs may play a similar regulatory function during viral infection.

In this study, a series of miRNA isomers (isomiR) were present in the miRNA expression profile. Although the phenomenon of isomiR variability is mainly due to the variability of the Dicer or Drosha cleavage positions on pre-miRNA hairpins, it may be related to the genomic variation under viral infection or tumorigenesis. Alternatively, different algorithms or different sequencing techniques may also lead to length and sequence heterogeneity of miRNAs [33, 34].

miRNA is known to be an extremely important part of the regulatory mechanisms of gene expression [35]. The expression profile and functional studies of miRNA have also revealed that miRNAs are involved in the regulation of almost all known cellular processes, and changes in miRNA expression levels may have a profound impact on the activities of the target genes [36, 37].

Although the role of miRNAs in cell development and tumorigenesis has been described in earlier studies, the importance of these small RNA molecules in the development and function of the immune system has only just begun to attract attention [38–40]. Recent studies have shown that miRNA expression is an early cellular response against exogenous or endogenous signal pressures. Additionally, to regulate the processes of specific immunity, miRNAs are also involved in the regulatory mechanisms of the innate immune response, including the proliferation of neutrophils and mononuclear cells [41]. The regulatory roles of miRNAs in immune processes include assisting in the development and differentiation of B and T lymphocytes and natural killer cells, and inducing the proliferation of mononuclear cells and neutrophils [42–44]. Therefore, the identification and characterization of the regulatory miRNAs involved in the immune response and exploration of their pathological roles in host-pathogen interactions are essential for the treatment and control of viral diseases.

Currently, widespread immunosuppressive viruses are causing huge losses to the farming industry. The discovery of a regulatory function for miRNAs in immunity provides us with a novel approach to prevent and treat viral diseases [45]. Pig is a major animal model for life science research; therefore, the discovery of a regulatory role for miRNAs during infection with an immunosuppressive virus in pigs should contribute to our understanding of how to overcome human infection with immunosuppressive viruses.

## Conclusion

In this study, we obtained the miRNA expression profile of PCMV-infected porcine macrophages by high-throughput sequencing. A total of 239 miRNA database-annotated and 355 novel pig-encoded miRNAs were detected. Of these, 130 miRNAs showed significant differential expression between the PCMV-infected porcine macrophages and the control samples.

## Supporting Information

S1 Fig Gene regulatory network formed by pig-encoded miRNAs (top 10) and their target genes in the pig genome. (PDF)

S1 Table Expression profile of porcine miRNAs. (XLS)

S2 Table Differentially expressed miRNAs in porcine macrophages. (XLS)

S3 Table Target prediction for differentially expressed miRNAs. (XLSX)

S4 Table GO functional enrichment annotations for the targets of the differentially expressed miRNAs. (XLSX)

S5 Table Real-time RT—PCR confirmation for miRNAs. (XLSX)

## References

[1] Gu W, Zeng N, Zhou L, Ge X, Guo X, Yang H. Genomic organization and molecular characterization of porcine cytomegalovirus. Virology. 2014; 460: 165–72. 10.1016/j.virol.2014.05.014 25010282

[2] Whitteker JL, Dudani AK, Tackaberry ES. Human fibroblasts are permissive for porcine cytomegalovirus in vitro. Transplantation. 2008; 86 (1): 155–62. 10.1097/TP.0b013e31817d4823 18622293

[3] Lee C-S, Moon H-J, Yang J-S, Park S-J, Song D-S, Kang B-K, et al Multiplex PCR for the simultaneous detection of pseudorabies virus, porcine cytomegalovirus, and porcine circovirus in pigs. J Virol Methods. 2007; 139 (1): 39–43. 17034871 10.1016/j.jviromet.2006.09.003

[4] Fryer JF, Griffiths PD, Fishman JA, Emery VC, Clark DA. Quantitation of porcine cytomegalovirus in pig tissues by PCR. J Clin Microbiol. 2001; 39 (3): 1155–6. 11230447 10.1128/JCM.39.3.1155-1156.2001 PMC87893

[5] Liu X, Liao S, Zhu L, Xu Z, Zhou Y. Molecular Epidemiology of Porcine Cytomegalovirus (PCMV) in Sichuan Province, China: 2010–2012. PloS one. 2013; 8 (6): e64648 10.1371/journal.pone.0064648 23762243 PMC3675093

[6] Clark DA, Fryer JF, Tucker AW, McArdle PD, Hughes AE, Emery VC, et al Porcine cytomegalovirus in pigs being bred for xenograft organs: progress towards control. Xenotransplantation. 2003; 10 (2): 142–8. 12588647 10.1034/j.1399-3089.2003.01128.x

[7] Mueller NJ, Kuwaki K, Dor FJ, Knosalla C, Gollackner B, Wilkinson RA, et al Reduction of consumptive coagulopathy using porcine cytomegalovirus-free cardiac porcine grafts in pig-to-primate xenotransplantation. Transplantation. 2004; 78 (10): 1449–53. 15599308 10.1097/01.tp.0000141361.68446.1f

[8] Fishman JA, Patience C. Xenotransplantation: infectious risk revisited. American Journal of Transplantation. 2004; 4 (9): 1383–90. 15307825 10.1111/j.1600-6143.2004.00542.x PMC7175990

[9] Ruby JG, Jan CH, Bartel DP. Intronic microRNA precursors that bypass Drosha processing. Nature. 2007; 448 (7149): 83–6. 17589500 10.1038/nature05983 PMC2475599

[10] Bartel DP. MicroRNAs: target recognition and regulatory functions. Cell. 2009; 136 (2): 215–33. 10.1016/j.cell.2009.01.002 19167326 PMC3794896

[11] Han J, Lee Y, Yeom K-H, Nam J-W, Heo I, Rhee J-K, et al Molecular basis for the recognition of primary microRNAs by the Drosha-DGCR8 complex. Cell. 2006; 125 (5): 887–901. 16751099 10.1016/j.cell.2006.03.043

[12] Alvarez-Garcia I, Miska EA. MicroRNA functions in animal development and human disease. Development. 2005; 132 (21): 4653–62. 16224045 10.1242/dev.02073

[13] Chen Z, Zeng H, Guo Y, Liu P, Pan H, Deng A, et al miRNA-145 inhibits non-small cell lung cancer cell proliferation by targeting c-Myc. J Exp Clin Canc Res. 2010; 29 (1):1. 10.1186/1756-9966-29-151 PMC2999592 21092188

[14] Huang S, He X. The role of microRNAs in liver cancer progression. Brit J Cancer. 2011;104 (2): 235–40. 10.1038/sj.bjc.6606010 21102580 PMC3031886

[15] Sempere LF, Freemantle S, Pitha-Rowe I, Moss E, Dmitrovsky E, Ambros V. Expression profiling of mammalian microRNAs uncovers a subset of brain-expressed microRNAs with possible roles in murine and human neuronal differentiation. Genome Biol. 2004; 5 (3): R13 15003116 10.1186/gb-2004-5-3-r13 PMC395763

[16] Válóczi A, Hornyik C, Varga N, Burgyán J, Kauppinen S, Havelda Z. Sensitive and specific detection of microRNAs by northern blot analysis using LNA-modified oligonucleotide probes. Nucleic Acids Res. 2004; 32 (22): e175–e. 15598818 10.1093/nar/gnh171 PMC545470

[17] Kozomara A, Griffiths-Jones S. miRBase: annotating high confidence microRNAs using deep sequencing data. Nucleic Acids Res. 2014; 42 (D1): D68–D73. 24275495 10.1093/nar/gkt1181 PMC3965103

[18] Malumbres R, Sarosiek KA, Cubedo E, Ruiz JW, Jiang X, Gascoyne RD, et al Differentiation stage—specific expression of microRNAs in B lymphocytes and diffuse large B-cell lymphomas. Blood. 2009; 113 (16): 3754–64. 10.1182/blood-2008-10-184077 19047678 PMC2670792

[19] Mathé EA, Nguyen GH, Bowman ED, Zhao Y, Budhu A, Schetter AJ, et al MicroRNA expression in squamous cell carcinoma and adenocarcinoma of the esophagus: associations with survival. Clin Cancer Res. 2009; 15 (19): 6192–200. 10.1158/1078-0432.CCR-09-1467 19789312 PMC2933109

[20] Linsen SE, de Wit E, de Bruijn E, Cuppen E. Small RNA expression and strain specificity in the rat. BMC Genomics. 2010; 11 (1): 249. 20403161 10.1186/1471-2164-11-249 PMC2864251

[21] Grey F, Meyers H, White EA, Spector DH, Nelson J. A human cytomegalovirus-encoded microRNA regulates expression of multiple viral genes involved in replication. PLoS Pathog. 2007; 3 (11): e163 17983268 10.1371/journal.ppat.0030163 PMC2048532

[22] Wu Y-Q, Chen D-J, He H-B, Chen D-S, Chen L-L, Chen H-C, et al Pseudorabies virus infected porcine epithelial cell line generates a diverse set of host microRNAs and a special cluster of viral microRNAs. PloS one. 2012; 7 (1): e30988 10.1371/journal.pone.0030988 22292087 PMC3264653

[23] Liu X, Xu Z, Zhu L, Liao S, Guo W. Transcriptome analysis of porcine thymus following porcine cytomegalovirus infection. PloS one. 2014; 9 (11): e113921 10.1371/journal.pone.0113921 25423176 PMC4244220

[24] Morin RD, O’Connor MD, Griffith M, Kuchenbauer F, Delaney A, Prabhu A-L, et al Application of massively parallel sequencing to microRNA profiling and discovery in human embryonic stem cells. Genome Res. 2008; 18 (4): 610–21. 10.1101/gr.7179508 18285502 PMC2279248

[25] Lu C, Shedge V. Construction of small RNA cDNA libraries for high-throughput sequencing cDNA Libraries: Springer; 2011: 141–52. 10.1007/978-1-61779-065-2_9 21365488

[26] Nobuta K, McCormick K, Nakano M, Meyers BC. Bioinformatics analysis of small RNAs in plants using next generation sequencing technologies Plant MicroRNAs: Springer; 2010: 89–106. 10.1007/978-1-60327-005-2_7 19802591

[27] Alexiou P, Vergoulis T, Gleditzsch M, Prekas G, Dalamagas T, Megraw M, et al miRGen 2.0: a database of microRNA genomic information and regulation. Nucleic Acids Res. 2009: gkp888. 10.1093/nar/gkp888 PMC2808909 19850714

[28] Consortium GO. The Gene Ontology (GO) database and informatics resource. Nucleic Acids Res. 2004;32 (suppl 1): D258–D61. 14681407 10.1093/nar/gkh036 PMC308770

[29] Scobie L. Porcine pathogens and xenotransplantation: PERV and beyond! Xenotransplantation. 2010; 17 (2): 118.

[30] Garzon R, Fabbri M, Cimmino A, Calin GA, Croce CM. MicroRNA expression and function in cancer. Trends Mol Med. 2006; 12 (12): 580–7. 17071139 10.1016/j.molmed.2006.10.006

[31] Li X, Zhu L, Liu X, Sun X, Zhou Y, Lang Q, et al Differential expression of micrornas in porcine parvovirus infected porcine cell line. Virol J. 2015; 12 (1):1. 26290078 10.1186/s12985-015-0359-4 PMC4545981

[32] Cai Y, Zhu L, Zhou Y, Liu X, Liu X, Li X, et al Identification and Analysis of Differentially-Expressed microRNAs in Japanese Encephalitis Virus-Infected PK-15 Cells with Deep Sequencing. Int J Mol SCI. 2015; 16 (1): 2204–19. 10.3390/ijms16012204 25608654 PMC4307358

[33] Guerau-de-Arellano M, Alder H, Ozer HG, Lovett-Racke A, Racke MK. miRNA profiling for biomarker discovery in multiple sclerosis: from microarray to deep sequencing. J Neuroimmunol. 2012; 248 (1): 32–9. 22078708 10.1016/j.jneuroim.2011.10.006 PMC3288464

[34] Jaskiewicz L, Filipowicz W. Role of Dicer in posttranscriptional RNA silencing RNA Interference: Springer; 2008 p. 77–97. 10.1007/978-3-540-75157-1_4 18268840

[35] van Rooij E, Sutherland LB, Qi X, Richardson JA, Hill J, Olson EN. Control of stress-dependent cardiac growth and gene expression by a microRNA. Science. 2007; 316 (5824): 575–9. 17379774 10.1126/science.1139089

[36] Dong H, Lei J, Ding L, Wen Y, Ju H, Zhang X. MicroRNA: function, detection, and bioanalysis. Chem Rev. 2013; 113 (8): 6207–33. 10.1021/cr300362f 23697835

[37] Shenoy A, Blelloch RH. Regulation of microRNA function in somatic stem cell proliferation and differentiation. Nat Rev Mol Cell Bio. 2014; 15 (9): 565–76. 25118717 10.1038/nrm3854 PMC4377327

[38] Rebane A, Runnel T, Aab A, Maslovskaja J, Rückert B, Zimmermann M, et al MicroRNA-146a alleviates chronic skin inflammation in atopic dermatitis through suppression of innate immune responses in keratinocytes. J Allergy Clin Immun. 2014; 134 (4): 836–47. e11 10.1016/j.jaci.2014.05.022 24996260

[39] Chan EK, Ceribelli A, Satoh M. MicroRNA-146a in autoimmunity and innate immune responses. Ann Rheum Dis. 2012: annrheumdis-2012-202203. 10.1136/annrheumdis-2012-202203 PMC7664460 23253933

[40] Cullen BR. MicroRNAs as mediators of viral evasion of the immune system. Nat Immunol. 2013; 14 (3): 205–10. 10.1038/ni.2537 23416678 PMC3642974

[41] Roy S, Sen CK. MiRNA in innate immune responses: novel players in wound inflammation. Physiol Genomics. 2011; 43 (10): 557–65. 10.1152/physiolgenomics.00160.2010 21139022 PMC3110889

[42] Li Q-J, Chau J, Ebert PJ, Sylvester G, Min H, Liu G, et al miR-181a is an intrinsic modulator of T cell sensitivity and selection. Cell. 2007; 129 (1): 147–61. 17382377 10.1016/j.cell.2007.03.008

[43] Fehniger TA, Wylie T, Germino E, Leong JW, Magrini VJ, Koul S, et al Next-generation sequencing identifies the natural killer cell microRNA transcriptome. Genome Res. 2010; 20 (11): 1590–604. 10.1101/gr.107995.110 20935160 PMC2963822

[44] Johnnidis JB, Harris MH, Wheeler RT, Stehling-Sun S, Lam MH, Kirak O, et al Regulation of progenitor cell proliferation and granulocyte function by microRNA-223. Nature. 2008; 451 (7182): 1125–9. 10.1038/nature06607 18278031

[45] Lee H-M, Nguyen DT, Lu L-F. Progress and challenge of microRNA research in immunity. Front Genet. 2014; 5:178 10.3389/fgene.2014.00178 24971086 PMC4053854

