# Supplementary figures and images for: Identification and Analysis of the Porcine MicroRNA in Porcine Cytomegalovirus-Infected Macrophages Using Deep Sequencing
Source: PLoS One. 2016 Mar 4;11(3):e0150971. doi: 10.1371/journal.pone.0150971 (PMC4778948; doi:10.1371/journal.pone.0150971)

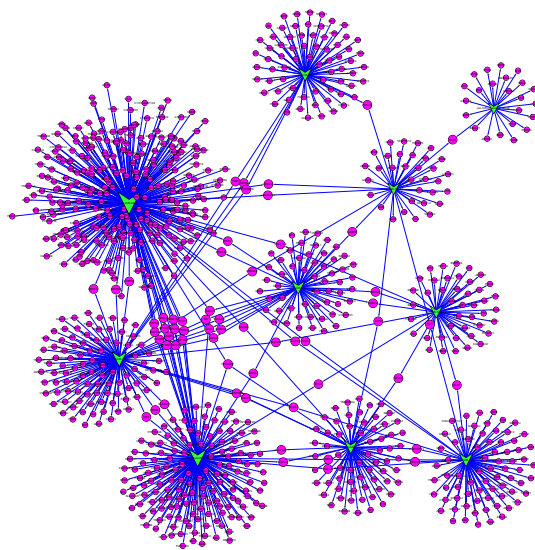

Supplement: S1 Fig — (PDF) [file pone.0150971.s001.pdf]
